# A Short-Term Physical Activity Randomized Trial in the Lower Mississippi Delta

**DOI:** 10.1371/journal.pone.0026667

**Published:** 2011-10-25

**Authors:** Peter T. Katzmarzyk, Catherine M. Champagne, Catrine Tudor-Locke, Stephanie T. Broyles, David Harsha, Betty M. Kennedy, William D. Johnson

**Affiliations:** Population Science, Pennington Biomedical Research Center, Baton Rouge, Louisiana, United States of America; Institut Pluridisciplinaire Hubert Curien, France

## Abstract

**Background:**

The purpose of this study was to determine if a short-term pedometer-based intervention results in immediate increases in time spent in moderate-to-vigorous physical activity (MVPA) compared to a minimal educational intervention.

**Methods:**

A sample of 43 overweight adults 35 to 64 years of age participated in a one week pedometer-based feasibility trial monitored by accelerometry. Participants were randomized into a one-week education-only group or a group that also wore a pedometer. Accelerometer-measured MVPA was measured over 7 days at baseline and again for 7 days immediately post-intervention.

**Results:**

Minutes of MVPA increased significantly in the overall sample (p = 0.02); however, the effect of adding the pedometer to the education program was not significant (p = 0.89). Mean (±SE) MVPA increased from 12.7±2.4 min/day to 16.2±3.6 min/day in the education-only group and from 13.2±3.3 min/day to 16.3±3.9 min/day in the education+pedometer group. The correlation between change in steps/day and change in MVPA was 0.69 (p<0.0001).

**Conclusions:**

The results of this study suggest that the addition of a pedometer to a short-term education program does not produce added benefits with respect to increasing physical activity in the Lower Mississippi Delta.

**Trial Registration:**

ClinicalTrials.gov NCT01264757

## Introduction

The Lower Mississippi Delta (LMD) population in the United States is characterized by high levels of poverty, food insecurity, obesity, and related chronic diseases. There is a need to identify new strategies that will enhance adherence to the healthful dietary and physical activity recommendations set forth in the *2005 Dietary Guidelines for Americans* [1] and the *2008 Physical Activity Guidelines for Americans* [2] in this population. In particular, more comprehensive knowledge of how to adapt federal dietary and physical activity recommendations for populations living in the LMD is desperately needed.

Of all types of physical activity, walking is the most commonly reported form of leisure physical activity [3], and it is also a functional component of shopping, transportation and other routine activities [4]. Thus, walking is a potentially valuable behavioral target when designing interventions to increase physical activity levels of the population. For residents of rural areas, interventions to increase physical activity via walking are particularly important as exercise facilities are less common than in more urban areas [5].

Pedometers are simple step-counting devices that can be used to assess levels of walking behavior in the population [6], [7], and are also useful in self-monitoring individual walking behaviors. Three recent meta-analyses reported that participants in pedometer-based interventions increased their physical activity and lost a modest amount of weight [8], [9], [10]. A recent study in the LMD demonstrated the feasibility of increasing average steps/day in a sample of primarily African American women over six months [11]. Although the change in overall pedometer-determined physical activity is promising, the degree to which these interventions were successful in increasing time spent in moderate-to-vigorous physical activity (MVPA) is not known, particularly in the LMD.

Louisiana has the fifth highest prevalence of adult obesity in the United States [12], and only 44% of adults in Louisiana report achieving 30 or more minutes of MVPA five or more days each week, or vigorous-intensity physical activity for 20 or more minutes during three or more days each week [13]. Thus, the purpose of this study was to determine, among community dwelling adults living in the LMD region of Louisiana: 1) if a pedometer-based educational intervention could elicit short-term, immediate increases in MVPA compared to a minimal education-only intervention, and 2) whether change in steps/day is associated with change in MVPA.

## Methods

The protocol for this trial and supporting CONSORT checklist are available as supporting information; see Checklist S1 and Protocol S1.

### Ethics Statement

Written informed consent was provided by all participants and the research protocol was approved by the Pennington Biomedical Research Center's Institutional Review Board.

### Overview

This was a randomized short-term (i.e., one week) pedometer-based physical activity feasibility trial, with outcomes measured by accelerometry. The primary outcome was changes in time spent in accelerometer-determined MVPA. Secondary outcomes include changes in accelerometer-determined steps/day, lifestyle activities and sedentary behavior. All assessments were conducted at baseline and immediately following the intervention.

### Participants

The sample included 43 adults from a small community in the Louisiana LMD region. The participants were recruited by local advertisements on the radio, newspaper, and flyers distributed to local stores. The inclusion criteria consisted of being 35 to 64 years of age, having a body mass index (BMI) between 25 and 34.9 kg/m^2^, and being able to walk without limitation. The exclusion criteria included having cardiovascular, respiratory, gastrointestinal, neuromuscular, neurological, or psychiatric problems; musculoskeletal problems interfering with exercise; immunodeficiency problems; malignancies in the last 5 years; or any other medical condition or life threatening disease that could be aggravated by exercise.

### Assessment

Height, weight and waist circumference were measured at baseline and immediately after the one-week intervention period. Height was measured using a portable stadiometer (Perspective Enterprises Model PE-AIM-101, Kalamazoo, MI) and weight was measured using a portable scale (Health-o-meter Professional Model 599KL, Boca Raton, FL), both without shoes. Waist circumference was measured at the midpoint between the iliac crest and the inferior margin of the rib cage using a standard clinician's measuring tape.

Time spent in MVPA, lifestyle activities and sedentary behavior were assessed using an ActiGraph Model GT3X accelerometer (ActiGraph, Pensacola, FL; formerly distributed as CSA, MTI, AM-7164) for one week at baseline and again for one week immediately post-intervention. Prior to distribution to participants, accelerometers were initialized to detect activity counts and steps in 1-minute epochs (i.e., time intervals). Participants were instructed to wear the device on the right hip using an elasticized belt and only to remove it at the end of each day and also during any water activities (e.g., swimming, showering, and bathing). Written instructions (that included space to record when the accelerometer was attached and removed each day) were sent home with the participant.

### Interventions

Participants were randomized equally into a one-week minimal education-only group or an education+pedometer group. Participants were randomly assigned to each intervention arm using a random group assignment produced by a computer in permuted blocks of n = 4 participants per block. The participant's group assignment was provided to the study co-ordinator by the biostatistician (WDJ), who in turn informed the study interventionists of the group assignment of the participant so they could schedule the intervention components accordingly. The education-only group received a brochure detailing the importance of physical activity for maintaining health, the physical activity guidelines, and strategies to increase physical activity levels. The education+pedometer group received the same educational materials, in addition to a YAMAX Digi-Walker SW-200 (made in Japan; distributed in the U.S. by New Lifestyles, Lee's Summit, MO) pedometer and instructions on its use. The participants were shown how to operate the pedometer, and walked outside with an interventionist for approximately 10 minutes to build self-efficacy for walking at MVPA and to observe how quickly steps accrued [14]. Specific strategies, like walking to lunch, walking the dog, parking farther from the worksite entrance, etc., were discussed during the 10 minute walk.

Participants were sent home with instructions to engage in usual activity for their first day of wearing a pedometer so as to become aware of their habitual step-determined physical activity. On subsequent days, they were to increase their steps/day by an amount that would approximate USDA guidelines for the prevention of weight gain [1]. Specifically, they were told that “a good guideline is to add 60 minutes of moderate intensity activity, like brisk walking, to your usual daily activities. This is equivalent to an additional 6000 steps. But it also depends on your baseline activity.” As a guide, participants were asked to consider taking an additional 6,000 steps if their habitual activity was less than 5,000 steps/day; to take an additional 5,000 steps if their habitual activity was between 5,000 to 7,500 steps/day; and if they were already taking more than 7,500 steps/day, to consider adding more steps and/or walking faster. Participants recorded their daily steps on a provided log sheet when the pedometer was attached and removed for the day.

### Data Treatment

Accelerometer data were downloaded on site and processed later at the research center. Daily time worn (hours and minutes) was computed using a SAS macro provided by the National Cancer Institute (NCI) at http://riskfactor.cancer.gov/tools/nhanes_pam/. A valid day was defined as having ≥10 hours of wear [15], [16] and we required a minimum of 3 valid days for determining time in MVPA [17]. Participants with fewer than 3 valid days of measurement at either baseline or follow-up assessment were excluded from analysis.

Each minute was classified as either sedentary behavior (<100 activity counts/minute), lifestyle activity (760–2019 activity counts/minute), MVPA (≥2,020 activity counts/minute), or other activity using the thresholds previously employed by studies analyzing NHANES data [15], [16], [18]. Daily records of time in sedentary behavior, lifestyle activities and MVPA were summed and divided by the number of valid days worn to determine average time spent in each category.

### Statistical Analysis

Differences between education+pedometer and education-only groups at baseline were tested using a Student's t-test. Time spent in sedentary activities, lifestyle actvities MVPA were summarized as group means (95% confidence intervals), separately for baseline and follow-up assessments. Due to skewed distributions in the baseline variables, descriptive results are also shown as medians (interquartile range). However, the distribution of change scores associated with the intervention did not appear to be skewed (visually and based on skewness statistics), and there was no evidence of asymmetry in the distribution. A repeated measures analysis of variance was employed to compare intervention groups with respect to changes in sedentary behavior , lifestyle activities and MVPA from the 7-day baseline (pre-intervention) assessment to the 7-day follow-up (post-intervention) assessment. Due to the skewed distribution of several variables at baseline, we also conducted a non-parametric analysis (median two-sample test); however, the results were essentially the same so we present only the ANOVA results. The relationships between change in steps/day, change in MVPA, change in lifestyle activities and change in sedentary time were assessed using Pearson correlation coefficients. All analyses were performed using SAS 9.1 (SAS Institute, Inc., Cary, NC), and the level for statistical significance was set at p<0.05.

## Results

**Figure 1:**
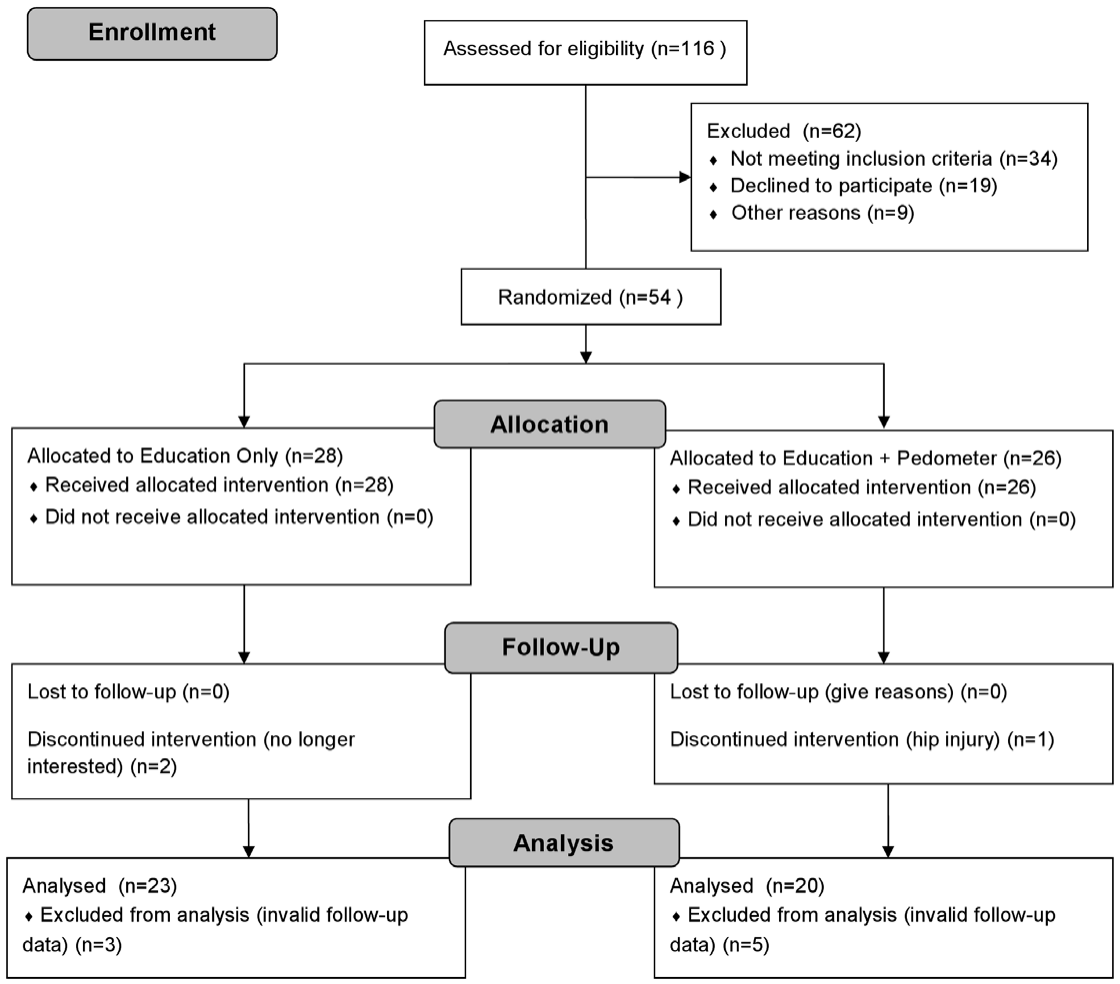
Flow of participants through the study.

The participant flow through the trial is presented in Figure 1. A total of 116 potential participants were screened, and 63 participants were interested in participating that met the eligibility criteria of the study. However, 9 participants failed to provide adequate baseline accelerometry data due to equipment malfunctions or having less than three days of at least 10 hours of wear time. After randomization, three participants dropped out of the study; two were no longer interested in participating, and one was unable to walk comfortably due to a pre-existing hip injury. Further, 8 participants failed to provide adequate accelerometry data at follow-up. Thus, the final sample size of participants with sufficient data for the planned analyses was 43. There were no significant baseline differences in age, BMI or waist circumference between the 43 participants who completed all study protocols versus those participants who did not.

The descriptive characteristics of the sample at baseline are presented in Table 1. There were no significant differences between the education-only and education+pedometer groups for any of the descriptive variables based on independent samples t-tests (Table 1). Further, a logistic regression analysis in which all baseline variables in Table 1 were simultaneously entered as predictors of group assignment (0 = education-only, 1 = education+pedometer) revealed that none of the variables has a significant association with group assignment after controlling for all other variables in the model.

**Table 1:** Descriptive characteristics of the participants at baseline.

	Education+Pedometer	Education-Only	p-value*
N	20	23	
Men (%)	20.0	13.0	.4396
White (%)	70.0	73.9	.7754
Age (y)	52.7 (8.8)	50.3 (7.7)	.3662
Weight (kg)	83.9 (14.0)	83.8 (13.2)	.9874
BMI (kg/m^2^)	30.8 (3.9)	31.6 (3.8)	.5202
Waist (cm)	96.7 (11.8)	97.1 (9.1)	.9056

*p-value for difference between groups.

**Figure 2:**
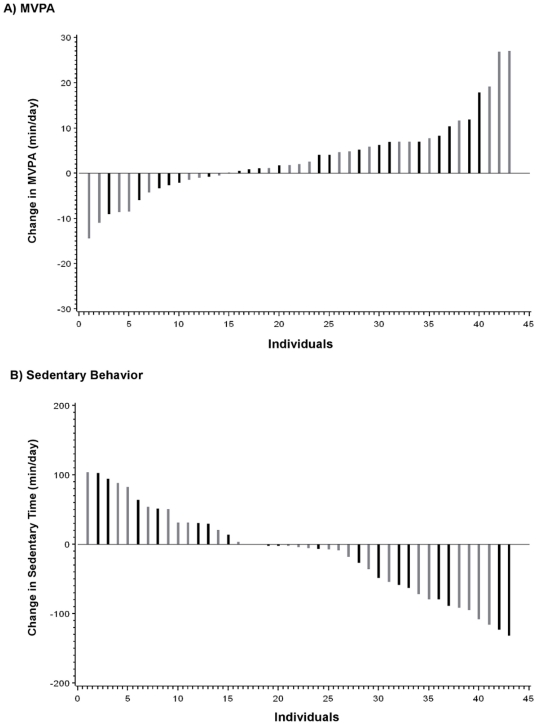
Individual changes in minutes of moderate-to-vigorous physical activity (MVPA) and time in sedentary behavior. Panel A presents results for MVPA and panel B presents results for sedentary behavior. Participants in the education-only group are represented by the gray bars, and those in the education+pedometer group are represented by the black bars.

On average, the sample can be considered to be “low-active” according to an established step-defined index of physical activity [19]. As expected, there were no significant changes in BMI (-0.002±0.07 vs −0.08±0.10 kg/m^2^) or waist circumference (−0.03±0.62 vs −0.06±0.76 cm) in the education+pedometer and education-only groups, respectively. Individual changes in minutes of MVPA and time in sedentary behavior for each participant in the two intervention groups are presented in Figure 2. The changes in MVPA ranged from a decrease of 14.4 min/day to an increase of 27.1 min/day, whereas the changes in sedentary behavior ranged from an increase of 104 min/day to a decrease of 131.6 min/day. The changes in lifestyle activity ranged from a decrease of 138.7 min/day to an increase of 86.0 min/day (results not shown).

**Figure 3:**
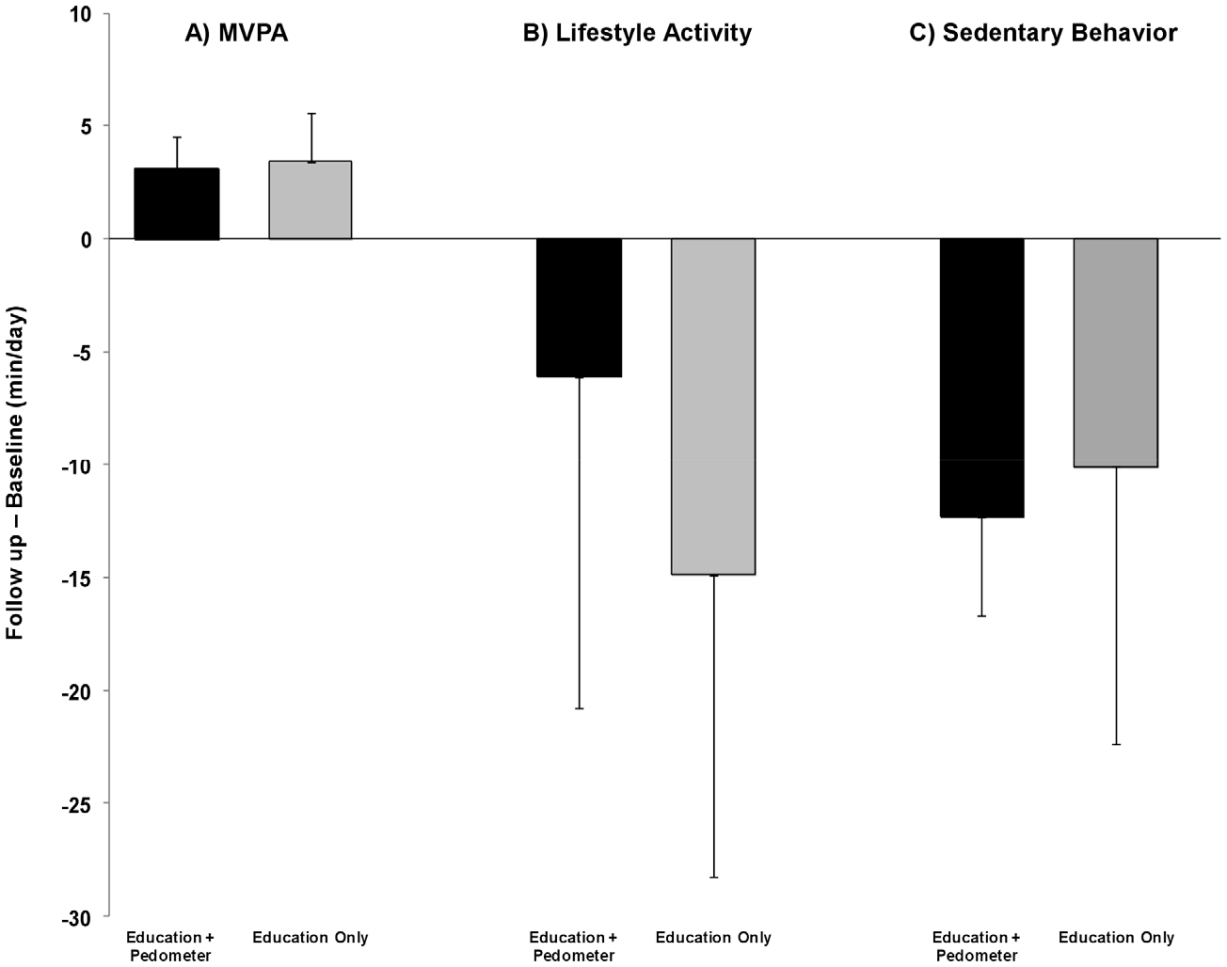
Changes in minutes of moderate-to-vigorous physical activity (MVPA), lifestyle activity, and time in sedentary behavior. Panel A presents results for MVPA, panel B presents results for lifestyle activity, and panel C presents results for sedentary behavior. The error bars represent 1 standard error.

**Figure 4:**
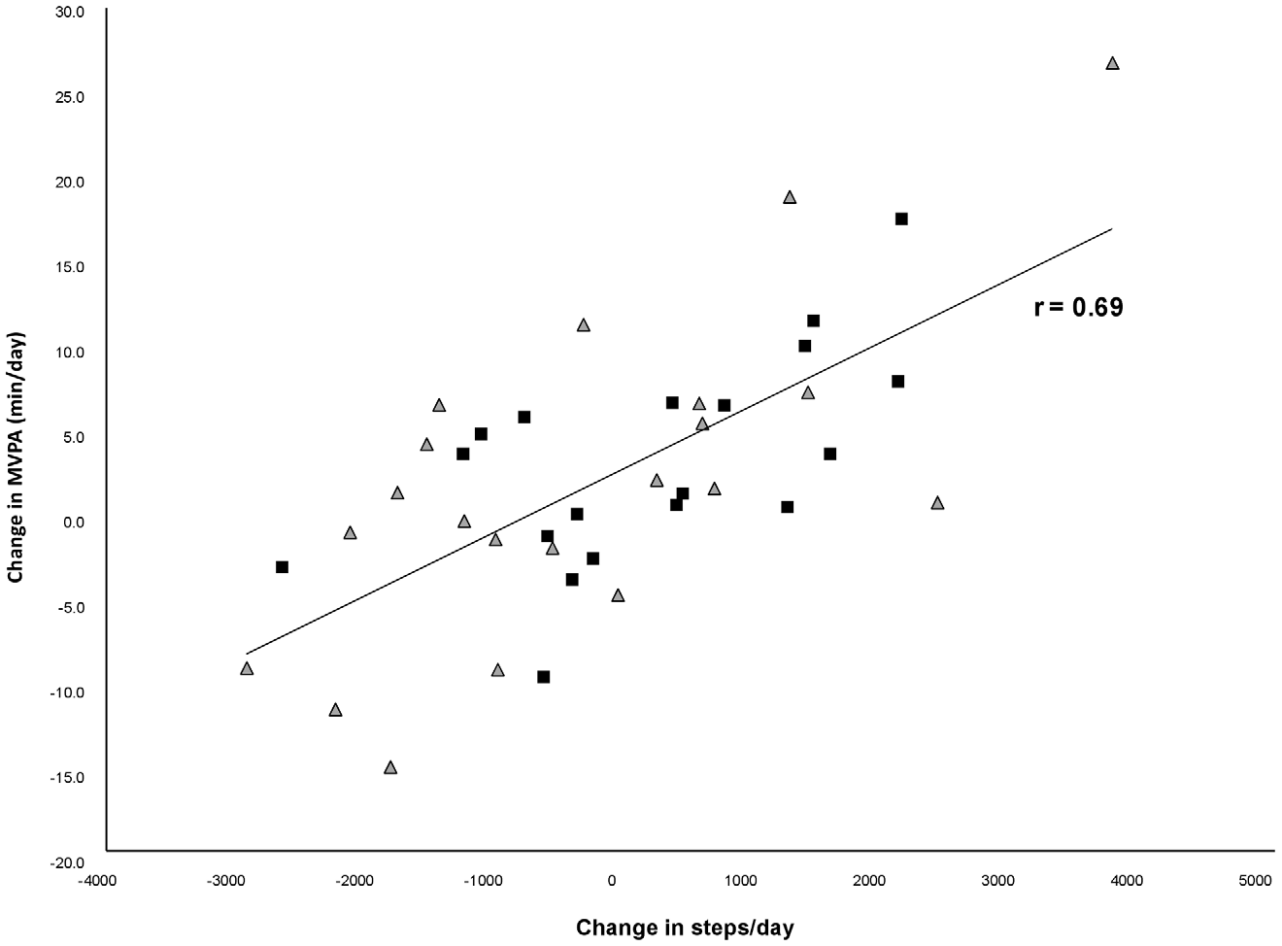
Scatter plot of changes in steps/day and changes in minutes of moderate-to-vigorous physical activity. The black boxes represent participants in the education+pedometer group and the gray triangles represent participants in the education-only group.

The group-specific mean and median minutes spent in MVPA, lifestyle activity and sedentary behavior at baseline and at follow-up are shown in Table 2. There was an overall increase in minutes in MVPA in the entire sample (p = 0.02); however, the effect of adding the pedometer was not significant (p = 0.89) (Figure 3). There was no overall increase in lifestyle activities (p = 0.14), and no effect of group assignment (p = 0.53) (Figure 3). Neither the overall decrease in sedentary behavior (p = 0.26), nor the difference between groups (p = 0.91) was significant (Figure 3). Similarly, neither the overall change in steps/day (p = 0.86), nor the difference between interventions (p = 0.26) was significant (Table 2). The correlations between changes in steps/day and changes in MVPA (min/day) were 0.69 (p<0.0001) in the overall sample (Figure 4), 0.72 (p = 0.0003) in the education-only group and 0.63 (p = 0.004) in the education+pedometer group. The correlations between changes in steps/day and changes in lifestyle activity (min/day) were 0.45 (p = 0.004) in the overall sample, 0.46 (p = 0.03) in the education-only group, and 0.48 (p = 0.04) in the education+pedometer group. The correlations between changes in steps/day and changes in sedentary behavior were −0.22 (p = 0.17) in the overall sample, −0.21 (p = 0.35) in the education-only group and −0.25 (p = 0.31) in the education+pedometer group.

**Table 2:** Accelerometer-determined steps/day, moderate-to-vigorous physical activity (MVPA), lifestyle activities and sedentary behavior in the education+pedometer and education-only groups at baseline and at follow-up.

		Education+Pedometer		Education-only	
		Baseline	Follow-up	Baseline	Follow-up
Steps/day	Mean (95% CI)	6836 (5747–7925)	7248 (5967–8529)	7113 (6028–8199)	6637 (5591–7682)
	Median (Interquartile range)	6961 (5002–8213)	7170 (5035–7930)	6729 (5564–9056)	5993 (4798–6888)
MVPA (min/day)	Mean (95% CI)	13.2 (6.3–20.2)	16.3 (8.2–24.4)	12.7 (7.8–17.7)	16.2 (8.8–23.6)
	Median (Interquartile range)	6.4 (3.3–22.2)	8.6 (4.3–23.9)	7.0 (4.0–18.7)	8.8 (4.8–26.3)
Lifestyle Activities (min/day)	Mean (95% CI)	74.4 (60.3–88.4)	68.3 (55.8–80.8)	99.2 (80.2–118.3)	84.4 (68.1–100.7)
	Median (Interquartile range)	69.2 (54.0–84.3)	70.2 (42.1–89.7)	92.6 (70.6–125.8)	65.7 (57.5–112.6)
Sedentary Behavior (min/day)	Mean (95% CI)	508 (464–552)	495 (454–537)	485 (450–519)	475 (440–509)
	Median (Interquartile range)	503 (430–574)	484 (423–576)	495 (434–541)	469 (422–524)

## Discussion

The results suggest that providing pedometers in addition to educational materials as an intervention to produce short-term changes in physical activity in LMD adults is not more effective than providing educational materials alone. Overall, there was a statistically significant (p = 0.02) increase in accelerometer-determined MVPA across both groups. Although the absolute magnitude of the increase was small - on average 3.3 min/day, this represents a relative increase of 25.5% from baseline. Thus, the study failed to achieve increases in MVPA that would approximate the current physical activity recommendations for the prevention of weight gain (60 min/day) or for the promotion of overall health (30 min/day) [1]. However, the observed relative increase (25.5%) in MVPA is not that different from the average increase in physical activity reported for other pedometer-based interventions of longer duration (26.9%) [9]. There was considerable inter-individual variability in response to the interventions employed in this study (Figure 2). It is clear that some individuals increased their MVPA and/or decreased their sedentary behavior substantially, while others experienced very little change in their behavior, as measured by the accelerometer.

The results of two meta-analyses suggest that on average, pedometer-based interventions result in modest increases of approximately 2000 to 2500 steps per day [8], [9]. In most of the studies reviewed, a pedometer was used both as a motivational tool and as the method of assessing the primary outcome (steps/day). Few studies have assessed changes in physical activity consequent to a pedometer-based intervention using accelerometers [20], [21], [22], [23], [24], and fewer still have examined the effects of interventions on MVPA [20], [21], [25]. Thus, limited information is available on changes in the amount of physical activity achieved at different intensities. By using an accelerometer to assess the primary outcomes, we were able to determine changes in sedentary behavior and MVPA as well as changes in overall steps/day. The use of accelerometers to measures changes in physical activity consequent to an intervention is becoming an accepted practice, as their outputs are considered sensitive to change [26]. The increase observed in MVPA indicates that there was an increase in the number of “purposeful” steps of at least moderate intensity, whereas the total number of steps may not have changed significantly, or even go down somewhat (although not significantly). Although the differences between the education+pedometer intervention and education alone were not significant, the results showed that changes in steps/day were correlated with changes in MVPA in both the education-only and education+pedometer interventions.

There is evidence that using a step-related goal such as 10,000 steps/day increases the probability that people will increase their overall physical activity within the context of a pedometer-based intervention [8], [9]. In a recent three-week study, 18 subjects were randomly assigned to either a group whose time in MVPA was self-monitored using an accelerometer (targeting 30 min/day MVPA) versus a group whose step counts were self-monitored with a traditional pedometer (targeting 10,000 steps/day). The investigators found that the group monitoring time in MVPA increased their time spent in MVPA more than the group monitoring step counts only [20]. Given the recent availability of user-friendly accelerometers, the potential exists for people to monitor their physical activity patterns at targeted intensities more directly than in the past. Identifying the best behavioral targets to promote adoption of MVPA is a fertile area for future research.

This study has several strengths and limitations. A major strength is use of accelerometers to objectively measure MVPA and sedentary behavior at baseline and at follow-up. This provided an impartial quantification of short-term changes in specific indicators of physical activity. However, due to differences in step functions between instruments (different pedometers, accelerometers, etc.), the changes observed in steps/day in this study may not be directly comparable to changes observed in other studies that have quantified changes using a pedometer. At least in the short term, there were no apparent benefits of adding a pedometer to educational materials. It remains possible that a study of longer duration would reveal significant differences in MVPA and sedentary behavior. An evaluation of the First Step Program showed that there were small increases in physical activity in the first two weeks of a behaviorally based pedometer intervention, but physical activity levels increased more significantly beginning in the third and fourth week of the intervention [27]. Therefore the present study needs to be extended to more clearly define the time-course of such health-enhancing behavior changes within the context of an intervention.

There was no true “control group” in this study, as it was designed to compare an education-only group to an education+pedometer group. Although this might be seen as a limitation, this design allowed for the determination of the short-term effects of the inclusion of the pedometer *per se*, separate from the educational component of the intervention. We see the design as an evolution beyond what has been already established in many pedometer-based intervention studies [9], [10]. Additionally, many randomized trials that have examined the effects of a pedometer on changes in physical activity have embedded the pedometer within a behavioral intervention, which typically also included group and individual counseling sessions, reminder phone calls, etc [9]. This approach makes it difficult to disentangle the specific effect of the pedometer from effects of the behavioral intervention. In the present study, the overall increase in MVPA was statistically significant but the effect of additionally including the pedometer was not significant. Similarly, a recent study in sedentary older women found that the provision of a pedometer did not produce greater increases in physical activity than a behavioral change program alone; however, there was less attrition reported in the group that received the pedometer [25]. Taken together, these results indicate that providing a pedometer may not elicit changes in physical activity beyond those elicited by the provision of educational materials or a behavioral change program.

In summary, the addition of a pedometer to a short-term physical activity education program did not produce increases in MVPA beyond those observed with the education program alone in this LMD population. However, participants who did increase their walking behavior had a propensity to also increase their time spent in MVPA. Lifestyle activities and sedentary behavior appeared to be unaffected by either intervention strategy. The study further demonstrates that effective assessment of ambulatory physical activity in the underserved Delta population can be achieved using accelerometers.

## Supporting Information

Protocol S1 (DOC)

Checklist S1 (DOC)
