# Supplementary material for: A Short-Term Physical Activity Randomized Trial in the Lower Mississippi Delta
Source: PLoS One. 2011 Oct 25;6(10):e26667. doi: 10.1371/journal.pone.0026667 (PMC3201968; doi:10.1371/journal.pone.0026667)
Supplement: Protocol S1 — (DOC) [file pone.0026667.s001.doc]

#
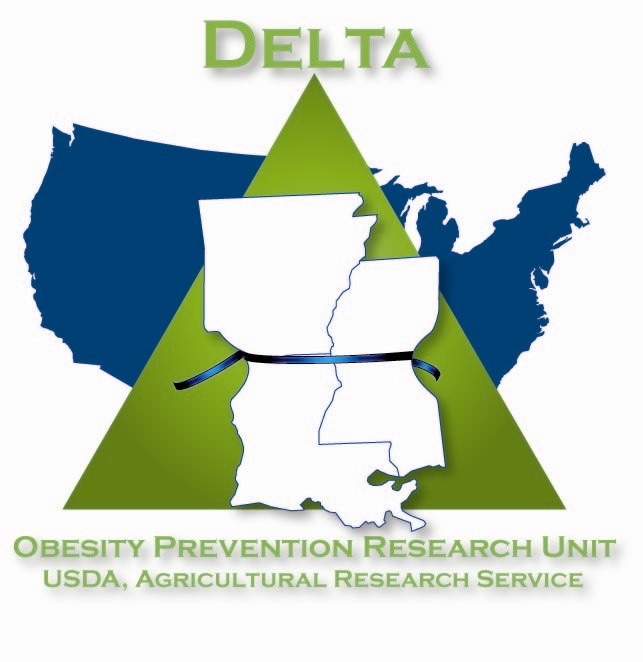


#
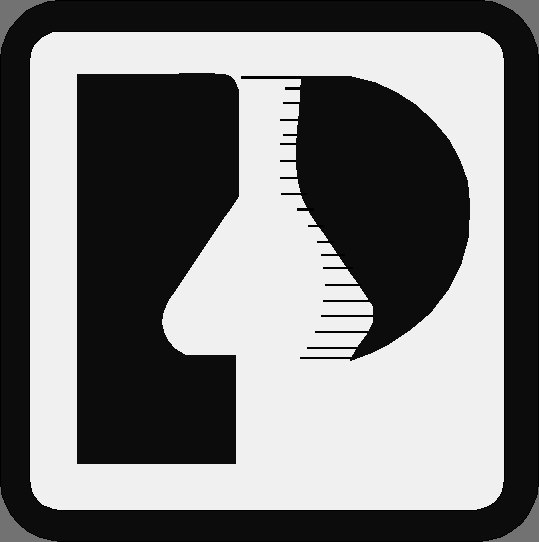


**Steps Toward Adapting Physical Activity Guidelines for the**

**Lower Mississippi Delta Population**

# Feasibility Study

# Protocol

**Pointe Coupee Parish, Louisiana**

**USDA, ARS**

**Pennington Biomedical Research Center**

###### TABLE OF CONTENTS

## Introduction ……………………………………………………………………….. 4

Goals and Objectives …………………………………………………………. 4

Overview of Feasibility Study………………………………………… 5

Study Procedures ………………………………………………………………. 5

## Baseline ………………………………………………………………. 5

Study Training …………………………………………………………. 5

Recruitment of Study Participants and Screening …………………… 5

Enrollment and Consent Procedure …………………………………… 5

Measurements …………………………………………………………. 6

General ………………………………………………………… 6

Dietary Assessment …………………………………………… 6

Anthropometric Assessment ………………………………….. 6

Physical Activity Assessment …………………………………. 8

Schedule of Measurements …………………………….. 12

Schedule of Intervention ………………………………… 13

Study Design ………………………………………………………. 14

## Data Management ………………………… 14

Statistical Analysis ………………………………………… 15

Expected Results ……………………………………………………………… 16

Human Subjects ………………………………………………………………. 16

Recruitment and Consent Procedures ………………………………… 17

Sources of Research Material …………………………………………. 17

Gender and minorities ………………………………………………… 17

Privacy ………………………………………………………… 17

Right to Know ………………………………………………… 17

DOPRU Research Team ……………………………………………………… 18

Study Timeline ……………………………………………………… 18

Appendix A. Study Flyer………………………………………………… 19

Appendix B. Initial Screening Form…………………………………… 21

Appendix C. Study Specific Questions………………………………… 24

# Appendix D. Automated Multiple Pass Methodology (AMPM)…......... 27

Appendix E. Physical Activity Readiness Questionnaire (PAR-Q)…… 31

# Appendix F. Physical Activity Education…………………………………..33

Study Forms …………………………………………………………………… 42

# Introduction

The Lower Mississippi Delta population is characterized by high levels of overweight and obesity, which are the result of long-term disruptions in energy imbalance where dietary energy intake has exceeded energy expenditure associated with physical activity. To address this issue, the overall goal of this project is to determine ways in which the *Dietary Guidelines for Americans, 2005* [DG] (U.S. Department of Health and Human Services and U.S. Department of Agriculture 2005) physical activity recommendations can be effectively adapted for the Lower Mississippi Delta population. In this study we will determine the feasibility of adapting physical activity recommendations by incorporating pedometers (step-counters) as self-monitoring tools within the context of an education program. Specifically, participants will be instructed to achieve DG physical activity recommendations and monitor the number of steps they take congruent with this success. Lessons learned from the feasibility study will be incorporated into the design of a future intervention in which we will conduct a properly powered, randomized controlled effectiveness trial of physical activity and adapted DG eating patterns to reduce unhealthy weight gain. An expectation from the short-term nature of the feasibility study is an immediate increase in physical activity (both number of steps and time in moderate to vigorous activity as assessed by accelerometer) based on the adopted use of a pedometer

The Pointe Coupee Parish community will be the targeted population for this study.

Goals and Objectives

The ultimate goal of this research is to achieve wider acceptance and use of the DG recommendations by the LMD population, leading to reduced prevalence of overweight and obesity and parallel reductions in the prevalence of chronic diseases and health care costs.

This feasibility study will specifically address the issue of adapting DG physical activity recommendations for the Delta population and examine the feasibility of adoption. A pedometer-based educational intervention using objective physical activity monitoring (accelerometers) will be used to assess the primary outcomes.

Hypothesis 1. Including a pedometer as a self-monitoring tool as part of adapted physical activity guidelines will result in greater immediate increases in physical activity (as measured by accelerometer) than an education program alone.

Hypothesis 2. Including a pedometer as a self-monitoring tool as part of adapted physical activity guidelines will result in a higher likelihood of achieving DG physical activity recommendations (as measured by accelerometer) than an education program alone.

Non-Hypothesis 3. Determine the number of steps (as measured by pedometer) associated with reaching the DG physical activity (as measured by accelerometer) recommendations.

**Overview of Feasibility Study**

A community site having adequate space for study measurements, completion of questionnaires and screening forms will be selected in Pointe Coupee Parish to serve as the study site for the feasibility study. The study will collect data on 60 eligible men and women residing in Pointe Coupee Parish, ages 40-64 years, and BMI of 25 to 29.9 kg/m^2^. Pointe Coupee Parish was chosen because it represents a typical LMD Parish in LA: a mix of rural and semi-rural populations, ethnically diverse (38% African American), and 26% of the population living in poverty (U.S. Census Bureau 2009a).

## **STUDY PROCEDURES**

**I) Baseline (Formative) Research.** During this time, we will publicly meet with community members to introduce the study. In addition, the intervention-site for the study will be determined.

**II) Study Training.** The investigators will provide training of staff for study implementation. At least one day of training will be conducted at PBRC in study procedures including protocol, consent, anthropometric measurements, dietary recalls, physical activity (including accelerometers and pedometers).

**III) Recruitment of Study Participants and Screening.** The goal for the feasibility study is to recruit a total of 60 males and females utilizing public service announcements (radio and TV), print (church bulletin announcements, and verbal announcements made by Pastors of churches in Pointe Coupee Parish during each church worship service, local community events, auxiliary meetings, word-of-mouth, presentations to various groups and organizations, and flyers (Appendix A) distributed at various businesses and churches throughout Pointe Coupee Parish. Recruitment and screening of study participants will be conducted using specific study criteria. A screening form designed to select eligible and exclude ineligible individuals will be utilized for the study. Recruitment for the feasibility study will begin on January 4, 2010 and end once 60 males and females are enrolled in the study. Baseline data collection will begin prior to the start of the study. We plan to conduct this study in Pointe Coupee Parish, LA. If we fail to achieve adequate sample sizes, we will begin recruiting subjects from adjoining Parishes, in particular, Avoyelles Parish.

**IV) Enrollment and Consent Procedure.** After screening, eligible subjects will be formally enrolled into the study. Each subject will be asked to sign an IRB-approved written informed consent form which details the purpose of study, the requirements for participation, and the potential benefits and risks. It will also indicate that participation is voluntary and may be terminated by the participant at any time. After the informed consent is obtained, baseline measurements will begin.

**V) Measurements.** All measurements will be conducted by designated staff. The following measurements will be made in all study participants as listed below:

1.General. During the recruitment phase all potential participants will complete an initial screening and study specific form to provide demographic, and health information such as age, past and present medical history. Initial screening form (Appendix B), and study specific form (Appendix C) are provided in the appendices noted.

2. Dietary Assessment. Dietary data will be collected at baseline by 24-hour recall (Appendix D)—using USDA’s Automated Multiple Pass Methodology (AMPM).

3. Anthropometric Assessment. Anthropometric assessment will consist of: measurements of height, weight, and waist circumference. With the exception of height, all other measures will be done at the beginning (baseline), and end of study. Height will be measured twice and averaged at baseline only without shoes to the nearest centimeter using a stadiometer. With the exception of height, weight will be measured twice and averaged at the beginning (baseline), and end of feasibility phase.

### **Height**

Height measurement to the nearest 0.1 cm is taken by a certified staff member using a stadiometer. Height is measured in inches with the participant standing on a firm, level surface that is at a right angle to the vertical board of the stadiometer.

Instruct the participant to remove shoes and headgear (hats and unusually large hairpieces) and to stand erect with feet flat on the floor and both heels together, touching the base of the vertical board. The participant stands erect with back, shoulder blades, and buttocks in contact with the vertical height board. If the participant cannot be positioned so that all of the above are in contact with the board, position so that the participant is standing erect with buttocks in contact with the board. The participant's weight is evenly distributed on both feet, and arms remain relaxed at the sides with palms facing inward. The participant stands facing straight ahead with his/her head in the horizontal (Frankfort) plane. The eyes of the examiner should be at the same level as the height indicator bar to obtain the most accurate measurement (Figure 1, Frankfort Horizontal Plane).

Ask the participant to inhale deeply and maintain a fully erect position without altering the load on the heels. Bring the height board down snugly, but not tightly, on the top of the participant's head. Record the height to the nearest 0.1cm.

**Figure 1. Frankfort Horizontal Plane for Measuring Body Height**


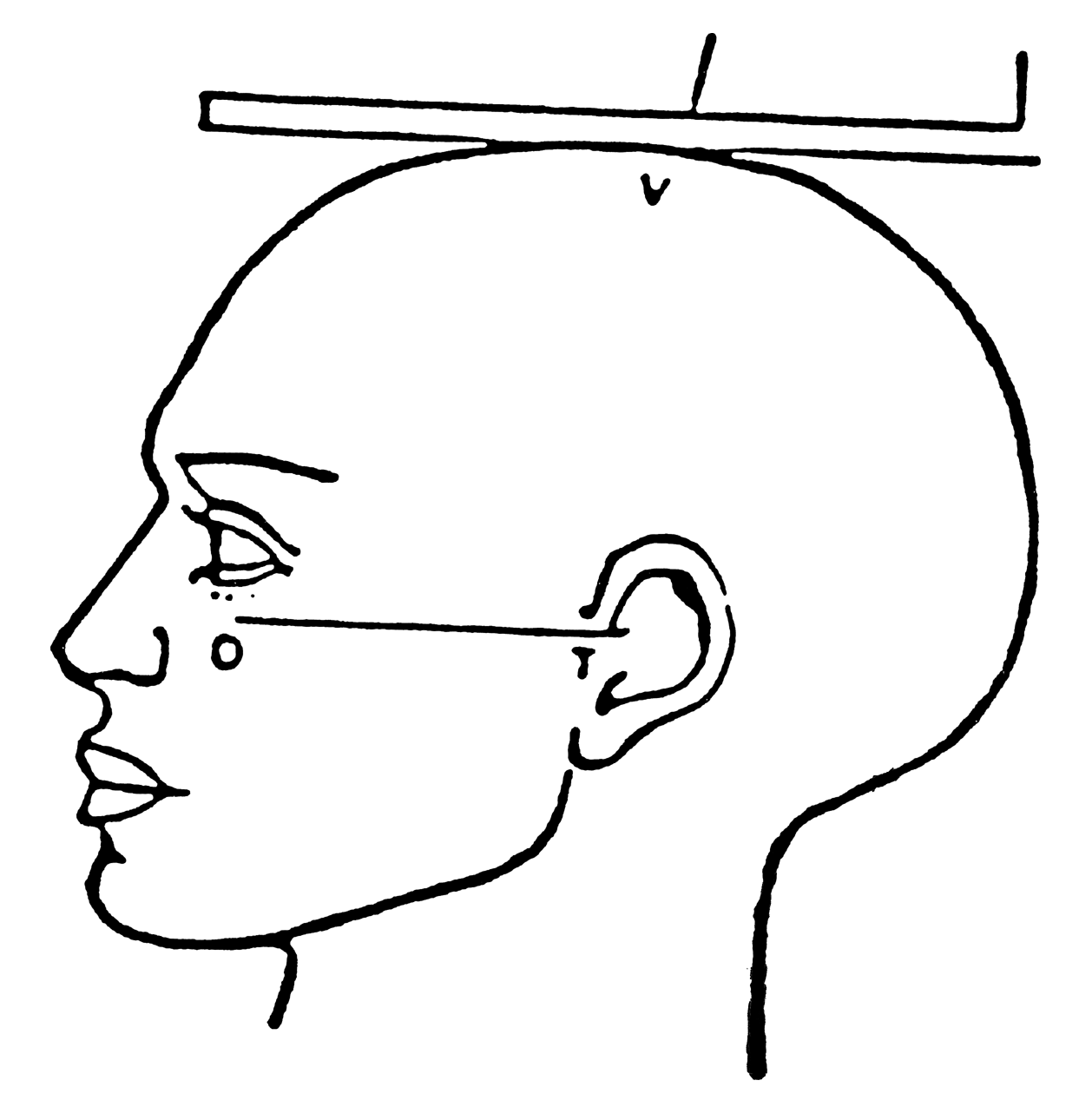


TRAGION: Notch above tragus of ear or at upper margin of zygomatic bone at that point.

FRANKFORT PLANE: Orbitale tragion line horizontal.

### **Weight**

Ask the participant to stand in the center of the scale platform, since standing off-center may affect the weight measurement. It is suggested that marks be made on the platform to insure the proper position of the participant's feet. The participant should stand with arms relaxed at the sides, head erect, and eyes looking straight ahead.

A Digital Medical Scale will be provided for weight measurements at the intervention study-site(s).

Make sure the scale reads "0" before the subject stands on the measurement platform. When the digital readout stabilizes, record the observed weight to the nearest 0.10 lbs or 0.25 kg.

All weight measurements consist of two independent weight assessments, and will be performed at baseline and end of study on each participant. Have the participant get off of the scale and then repeat the above procedure and record both weights. The computer will determine the official weight measurement after data entry. In the meantime, staff should report the approximate weight, to the nearest whole pound, to the participant.

**Waist Circumference**

**Procedure**

Instruct the participant to gather his or her shirt above the waist, cross the arms, and place the hands on opposite shoulders. Demonstrate the desired position of the arms. It may help to tell participants to think of giving themselves a hug. If necessary, lower the pants and underclothing to slightly below the waist. Again, always tell the participant what you are going to do before you do it.

Stand on the participant’s right side. Palpate the hip area to locate the right ilium of the pelvis. With the cosmetic pencil draw a horizontal line just above the uppermost lateral border of the right ilium. Cross this mark at the midaxillary line, which extends from the armpit down the side of the torso. Exhibit 3-9 shows the measurement site correctly marked for the waist circumference.

Extend the measuring tape around the waist. Position the tape in a horizontal plane at the level of the measurement mark, and ensure the horizontal alignment of the tape. Check that the tape sits parallel to the floor and lies snug but does not compress the skin. Always position the zero end of the tape below the section containing the measurement value. Take the measurement to the nearest 0.1 cm at the end of the participants.

4. Physical Activity Assessment. Study participants will be asked to respond to a physical activity readiness questionnaire (PAR-Q) [Appendix E) for safety reasons before beginning any physical activity regime. Accelerometers and Pedometers will be provided and used to measure physical activity and to increase steps.

Acceleromter Procedure

The ActiGraph accelerometer will be used to determine physical activity at baseline and at follow-up as follows:

The measurement of physical activity pattern and volume, specifically minute-by-minute participation in different intensities of physical activity. The equipment used is the ActiGraph GT3X manufactured by ActiGraph, of Ft. Walton Beach, FL ([www.theactigraph.com](http://www.theactigraph.com/)), and previously distributed as the GT1M, the 7164, the MTI, and the CSA. An ActiGraph User Manual is also available for download from this website.

Process Overview

1. Charging accelerometer
2. Initializing accelerometer (preparing the device to collect data)
3. Instructing the participant on self-monitoring procedures
4. Downloading data
5. Preparing electronic files for archiving

Procedure

1. Charging accelerometer

| - Research Nurse/Coordinator - Clinic Staff | - 1. Connect the GT3X to a standard computer USB port using the USB cord provided. Allow time for recharging. Charging time will depend on battery life but will not usually exceed 3 hours for a fully depleted battery. The GT3X will issue a single LED flash to indicate it is charging while plugged into computer.   2. Charging is complete when the LED is steady on while plugged into the computer. |
| --- | --- |

1. Initializing accelerometer (preparing the device to collect data)

| - Research Nurse/Coordinator - Clinic Staff | - 1. Initialize the accelerometer before the subject arrives.   2. If there is no desk top icon for the ActiLIfe Software, open the program through the “Start” menu by navigating to “All Programs -> Actigraph.”   3. Once the ActiLife start screen is viewable, click the Start button.   4. Plug in the GT3X to the designated USB port using the USB cord providing. Use this same USB port always for initialization and download.   5. When device is recognized, Click OK to continue.   6. On the initialization screen click GT3X Mode, Activity, Step Count, and Enable Stop Time.   7. Select a 60 second epoch.   8. Enter start date time to be the next day at 4:00am.   9. Enter the stop date time to be 7 days after the start day at 4:00am.   10. Enter the subject identifier in the subject information cell.   11. Select OK   12. Wait while initialization proceeds.   13. Disconnect the device from the computer and click OK.   14. Select an appropriately sized elastic belt (24, 36, 48, or 60 inches).   15. Lace the belt through the back of the provided pouch.   16. Place the initialized device in the pouch with the word ActiGraph at the bottom of the pouch.   17. The unit is now ready to collect data. |
| --- | --- |

1. Instructing the participant on self-monitoring procedures

| - Research Nurse/Coordinator - Clinic Staff | - 1. Demonstrate how to wear the belt on the waist. Align the ActiGraph over the right hip (mid axillary line). The unit can be worn either above or below clothing. It is not necessary for it to make contact with the skin.   2. Instruct the subject to wear the accelerometer during waking hours for 7 days, engage in usual activities, and remove the device only during any water activities (e.g., swimming, showering, and bathing)   3. Instruct them not to otherwise tamper with the device. |
| --- | --- |

1. Downloading data

| - Research Nurse/Coordinator - Clinic Staff | - 1. Choose the same computer and USB port that was used when the device was first initialized.   2. If there is no desk top icon for the ActiLIfe Software, open the program through the “Start” menu by navigating to “All Programs -> Actigraph.”   3. Click on the Download button on the start screen. If the device is not connected to the original USB port the software will indicate so.   4. Wait for downloading to complete.   5. Once complete, Click Yes to save data to file. The default destination will be C: Program Files\ActiGraph\ActiLife\Files |
| --- | --- |

1. Preparing electronic files for archiving

| - Research Nurse/Coordinator - Clinic Staff | - 1. Choose the same computer and USB port that was used when the device was first initialized and subsequently downloaded   2. If there is no desk top icon for the ActiLIfe Software, open the program through the “Start” menu by navigating to “All Programs -> Actigraph.”   3. Click on Analyze Data on the Menu bar.   4. Select Create Graphs.   5. MS Excel will load. Click on the graphic in the center of the Excel document.   6. Select the specific data file (.DAT) file on which the analysis is to be performed. It should be identifiable by the subject identifier.   7. Confirm that Text, Graph, and Cutlines are selected for Activity. Confirm that Text and Graph are selected for Step Count. Leave the Graph Titles blank.   8. Click continue. Save the output as Subjectidentifiergraphs.xls.   9. Return to the ActiLife software.   10. Click on Analyze Data on the Menu bar.   11. Select Create Caloric Output File   12. Confirm Freedson Equation is selected.   13. Select the specific data file (.DAT) file on which the analysis is to be performed. It should be identifiable by the subject identifier.   14. Name the output to be saved as Subjectindentifiercalories.csv   15. Select kilograms and enter the subject’s mass (weight) in kilograms.   16. Confirm that an MS Excel File has loaded with the name Subjectindentifiercalories.csv   17. Close the file.   18. Arrange for transfer of both files to RCG. |
| --- | --- |

A pedometer will be used in this study to encourage participants to increase their walking behavior. We will employ the Lifestyles NL1000 pedometer.

Pedometer NL 1000 Procedure

The measurement of physical activity pattern and volume, specifically steps/day and time in moderate+ activity. The equipment used is the NL-1000 distributed by New Lifestyles. An NL-1000 User Manual is also available for download (http://www.new-lifestyles.com/NL-1000_Users_Guide.pdf).

Process Overview

1. Installing the battery
2. Set modes for measurement
3. Instructing the participant on self-monitoring procedures
4. Recording data

Procedure

1. Installing the battery

| - Research Nurse/Coordinator - Clinic Staff | - 1. Use a coin to pop open the battery cover.   2. Identify the top “+” side of the battery   3. Insert the battery with the “+” side facing up .   4. Replace the battery case cover.   5. A low charge icon will appear on the digital screen when battery power is low and needs to be replaced |
| --- | --- |

1. Set modes for measurement

| - Research Nurse/Coordinator - Clinic Staff | - 1. Enter the set mode manually by pressing and holding the SET button for 3 seconds.   2. You will begin with time of the day. Press the + button until the flashing digits reflects the correct hour. Use the – button to back up. Pay attention to AM or PM to set correct time.   3. Press SET to move on to minutes. Repeat the process to set minutes.   4. Leave stride length at default 2’ 6”. Press SET to move to Real-time Display Mode. |
| --- | --- |

1. Instructing the participant on self-monitoring procedures

| - Research Nurse/Coordinator - Clinic Staff | - 1. Demonstrate how to wear the pedometer horizontally on the waist band (half way between navel and hip). It is not necessary for it to make contact with the skin. It must be horizontal (not rotated). Use the provided security strap.   2. Instruct the subject to take 20 steps and check the pedometer. It should say 19-21 steps, else move its attachment to insure correct placement (not rotated).   3. Instruct the subject to wear the pedometer during waking hours for 7 days, engage in usual activities, and remove the device only during any water activities (e.g., swimming, showering, and bathing)   4. Instruct them not to otherwise tamper with the device.   5. Instruct them on how to complete the recording form (date, time on, time off, day end steps). Ask them to record time that the instrument was removed.   6. This pedometer does not require resetting at the end of the day. It automatically resets at midnight and records the previous day’s value in its memory. |
| --- | --- |

1. Recording data

| - Research Nurse/Coordinator - Clinic Staff | - 1. When the pedometer and written record are returned quickly scan for unusual < 1000 steps or >20000 steps in a day) and missing values. Query unusual values and make notes in the Comments column.   2. When subject has left, scroll through memory function to verify written records. Press the Memory button. When indicator arrow is on Steps, two pieces of alternating flashing data are available: the day and the number of steps for that day. Day = 1 is yesterday, Day =2 is two days ago, etc. Wait for the steps accumulated for each day to flash, then press Memory to advance to the next day. Note discrepancies in the Comments column.   3. Arrange for transfer of records to RCG. |
| --- | --- |

**Study Measurement Schedule**

| **Measurements** |  | | |
| --- | --- | --- | --- |
| **Week ⇒** | Baseline | **2** | **3** |
| Initial Screening | • |  |  |
| Study specific questions | • |  |  |
| Height | • |  |  |
| Weight | • |  | • |
| Waist circumference | • |  | • |
| 24-Hour Recall (AMPM) | • |  |  |
| Physical activity(PAR-Q) Pedometers | • |  |  |
| Accelerometry |  |  | • |
| Pedometry |  | • | • |
| Post-study survey |  |  | • |

# Intervention schedule

| **Days →** | 1 | 2 | 3 | 4 | 5 | 6 | 7 | 1 | 2 | 3 | 4 | 5 | 6 | 7 | 1 | 2 | 3 | 4 | 5 | 6 | 7 |
| --- | --- | --- | --- | --- | --- | --- | --- | --- | --- | --- | --- | --- | --- | --- | --- | --- | --- | --- | --- | --- | --- |
| **Intervention group**  Accelerometers distribute/return | • |  |  |  |  |  | • |  |  |  |  |  |  |  | • |  |  |  |  |  | • |
| Education materials |  |  |  |  |  |  |  | • |  |  |  |  |  |  |  |  |  |  |  |  |  |
| Pedometers distribute/return |  |  |  |  |  |  |  | • |  |  |  |  |  | • | • |  |  |  |  |  | • |
|  | | | | | | | | | | | | | | | | | | | | | |
| **Control group**  Accelerometers distribute/return | • |  |  |  |  |  | • |  |  |  |  |  |  |  | • |  |  |  |  |  | • |
| Education materials |  |  |  |  |  |  |  | • |  |  |  |  |  |  |  |  |  |  |  |  |  |

**Intervention Phase**

The study will take place over a period of 30 days. After randomization, participants will be assigned to either the intervention or control group. Participants will be asked about their usual diet. They will then be asked to wear an accelerometer (a device that measures physical activity and inactivity) for 7 days (one week). When they return, they will be asked about their physical activity. The next 7 days (one week), both groups will receive culturally tailored educational materials (Appendix E) about how to become physically active. Participants assigned to the intervention group will receive a pedometer along with directions about how to use it to help increase their physical activity. They will be asked to write down the number of steps taken each day they wear the pedometer. The last 7 days (one week), the intervention group will be asked to wear the accelerometer again and also a pedometer. The control group will be asked to only wear the accelerometer again. When participants in the intervention group return the accelerometers, they will be asked about their experiences over the previous few weeks. Directions and personal instructions will be provided on how to use the pedometer as a self-monitoring tool to increase the number and intensity of steps participants take each day to increase the probability of meeting the DG physical activity recommendations. Examples will be provided of walking paces that correspond to moderate-to-vigorous physical activity to help ensure that walking is undertaken at a suitable intensity**.** Self-monitoring will be enhanced by having participants keep a daily log of the number of steps they take each day when they rest their pedometer.

**Limitations of the Intervention**

The potential limitation and/or problems to be anticipated include non-compliance and loss to follow-up. To minimize this effect, Walmart gift card in the amount of $30 will be given to all study participants. In addition, all participants will receive a pedometer to keep as an incentive.

Study Design

We will conduct a randomized controlled trial to determine the feasibility of using pedometers to cause immediate short-term increases in moderate-to-vigorous physical activity in the Lower Mississippi Delta population.

#

Subjects

# A total of 60 adults (men and women 35 - 64 years of age) living in Point Coupee Parish, Louisiana will be recruited and randomized to either the control or intervention group.

**Data Management**

Each participant will be issued an assigned ID number that will be utilized throughout the intervention. A secure master file linking names, addresses and ID numbers will be maintained in a confidential computer file accessible only to the Principal Investigator. Access to data files can be made only with permission of the Principal Investigator. The nutritional epidemiology, biostatistics, and computer administration sections at the Pennington Biomedical Research Center will provide support for data collection and management, including data forms, on-site data management and data transfer. Privacy in the context of this study includes confidentiality of data and personal information. During interviews and measurements, the intervention staff will ensure full privacy of participants and will ensure that the data are stored in a secured area. All intervention staff must be HIPAA certified. Only trained and certified examiners will collect data. The PBRC Biostatistics Department will cooperate with Ms. Connie Murla, the data manager, who will manage the data entry into the clinical database to ensure quality of data.

**Randomization Process:**

Each eligible volunteer who is accepted into the study will be randomized to one of two levels of treatment. Randomization will be done by the PBRC Biostatistics group and results will be stored in the study database and transmitted back to the investigators.

**Data Entry**

Double entry will be used for all manual entry of data to help ensure the fidelity of the database; scanable forms used wherever possible to minimize the need for manual data entry. The PBRC Data Management Group will have the prime responsibility for database design and implementation, programming, coordination and manual data entry of the study data into the study database.

**Statistical Analysis**

Analyses will be performed using SAS software. In the case of missing data, several approaches will be used, including an analysis restricted to participants with complete data; a last observation carried forward analysis, and a mixed model analysis. Sensitivity of analytical results to these alternative approaches will be assessed. Statistical tests that result in p-values ≤0.05 will be considered indicative of statistical significance.

The focus of the statistical analysis for **Hypothesis1** will be on comparing intervention and control groups with respect to changes in accelerometer-measured physical activity from the 7-day baseline (pre-intervention) assessment to the 7-day follow-up (post-intervention) assessment. Physical activity (minutes of moderate-to-vigorous physical activity) will be summarized as group means and 95% confidence intervals, separately for baseline and follow-up assessments. Student’s t-test will be used to assess statistical significance of differences in group means for individual physical activity change scores = post-score ─ pre-score. Although this is a feasibility study, we feel that a clinically significant increase in number of daily minutes of moderate-to-vigorous physical activity in this sedentary population would be a 100% increase.

The focus of the statistical analysis for **Hypothesis 2** will be on comparing intervention and control groups with respect to possible increase in percentage of participants achieving DG physical activity recommendations. The (nonparametric) permutation test will be used to assess differences in change in percent achievers in intervention vs. control groups (the sample sizes in this feasibility study are not sufficient to justify employing chi-square tests). Although this is a feasibility study, we feel that a clinically significant increase in the proportion of participants achieving the DG physical activity recommendations will be a doubling of the baseline proportion.

The focus of the statistical analysis for **Non-Hypothesis 3** will be on determining the number of steps associated with reaching the DG physical activity recommendations of 60 minutes a day of moderate-to-vigorous physical activity. General linear mixed models will be used to assess the cross-sectional relationship between daily number of steps (from pedometer) and minutes of moderate-to-vigorous physical activity (from accelerometer) using data collected post-intervention on all subjects in the intervention group. A mixed model approach is required to incorporate all seven days worth of data collected on each participant. The number of steps corresponding to 60 minutes of moderate-to-vigorous physical activity will be determined, and potential gender differences in the target number of steps will be examined. If significant gender differences exist, gender-specific step targets will be calculated and used in the future effectiveness study.

## **EXPECTED RESULTS**

- Increased knowledge,
- Positive behavior change, and
- Improved health effects in the short and long term.

The new knowledge obtained from this research will inform research, public policy and public health efforts to prevent obesity and its related disorders in the LMD. The results of this project will produce innovative models for understanding the influences on behaviors impacting energy imbalance and procedures for promoting changes in these variables. This information will allow national, regional, state, and local policy makers to better prioritize the use of resources to address the obesity epidemic in the LMD. Viewed from a wider perspective, the results from the studies in this population will inform other researchers in areas of the United States working with rural populations who also present with high levels of poverty and issues regarding food access and availability and problems achieving recommended physical activity recommendations.

# HUMAN SUBJECTS

The inclusion and exclusion criteria for the feasibility study are designed to maximize the number of participants who are eligible to participate, while maintaining the safety of the participants and the ability of the feasibility study to inform the development of the effectiveness trial in Phase II of the research project.

# Inclusion Criteria

Male or female living in Pointe Coupee Parish and:

- age 35 -64 years
- body mass index (BMI) 25 to < 35 kg/m^2^
- physically capable of undertaking physical activity
- have the capability and willingness to give written informed consent,
- understand exclusion criteria, and
- accept the randomized group assignment

**Exclusion Criteria**

The Study Physician, Dr. Timothy Church, and Principal Investigator may exclude individuals from the study for any or no reason.

A past history and/or physical examination or laboratory findings of a medical condition including (but not limited to):

- - Cardiovascular, respiratory, gastrointestinal, neuromuscular, neurological, or psychiatric conditions.
  - Musculoskeletal problems interfering with exercise.
  - Immunodeficiency diseases or a positive HIV test.
  - Malignancies in the past 5 years, with the exception of skin cancer therapeutically controlled.
  - Any other medical condition or disease that is life-threatening or that can interfere with or be aggravated by exercise

**Recruitment and consent procedures.** The Institutional Review Board and the Data Safety Monitoring Board will approve all procedures and study protocol at the Pennington Biomedical Research Center. Upon enrollment, each participant will be asked to sign an approved written informed consent form which details the purpose of the study, requirements for participation, and the potential benefits and risks. It will also indicate that participation is voluntary and may be terminated at any time.

**Sources of research material.** The procedures for obtaining research materials will include interviews, and questionnaires. Trained and qualified staff will perform these tasks. Data will be confidentially collected directly from study participants, to be used specifically for research purposes. All information and response to intervention will be documented in each participant’s permanent record with a unique identification number. No identifying information will be released from the study site. All information will be treated as confidential.

**Gender and minorities.** The study population will consist of adults (male and female) of all races, age 40-64 years. Children and adolescents will not be included in this study because the intervention strategy and materials are not

appropriate for this age group.

**Privacy.**  Privacy in the context of this study includes confidentiality of data and personal information. During interviews and measurements, the study staff will ensure full privacy of participants and will ensure that the data are stored in a secured area.

**Right-to-know.** All information obtained from each participant will be available to that participant.

## **DOPRU RESEARCH TEAM**

**Principal Investigator:**

Peter Katzmarzyk, PhD

**Medical Investigator:**

Timothy Church, MD

**Co-Investigators:**

Catherine Champagne, PhD, RD

Catrine Tudor-Locke, PhD

William Johnson, PhD

Stephanie Broyles, PhD

David Harsha, PhD

Betty Kennedy, PhD

**DOPRU 30-day Feasibility Phase I**

**TIMELINE***

| **Date** | **Activity** |
| --- | --- |
| January 4, 2010 | Meet with community/Recruitment begins |
| January 7, 2010 | Preliminaries/Training of staff, etc. |
| January 14, 2010 | Screening/enrollment first 7 days |
| January 21, 2010 | Feasibility study begins |
|  |  |

## ***Subject to change**

###### Appendix A

Study Flyer

**The Pennington Biomedical Research Center**

**Wants YOU!**

**The Pennington Center is planning to conduct a study**

**in**

**Pointe Coupee Parish**

**to determine ways in which**

**physical activity can be introduced to the**

**Lower Mississippi Delta community.**

**Participants must be between**

**the ages of 35 - 64,**

**a little overweight, and capable of**

**engaging in physical activity.**

**Other exclusions apply and**

**will be discussed in detail**

**if you have an interest in**

**taking part in this research study.**

**For more information contact**:

Pennington Biomedical Research Center

(225) 763-3090

(225) 763-3045 FAX

Email: Betty.Kennedy@pbrc.edu

###### Appendix B

Initial Screening Form

| Steps to Increase Physical Activity  INITIAL SCREENING |  |
| --- | --- |

Study Name: Steps To Increase Physical Activity Study Number:

Last Name: | | | | | | | | | | | | | | | | | | | |

First Name: | | | | | | | | | | | | | | | | | Middle Initial | |

______________________________________________________________________________________

Mailing Address: | | | | | | | | | | | | | | | | | | | | | | | | |

Apartment: | | | | | Zip Code: | | | | | |

City: | | | | | | | | | | | | | | | | | | | | State: | | |

______________________________________________________________________________________

Home Telephone Number: | | | | - | | | | - | | | | |

Work Telephone Number: | | | | - | | | | - | | | | | Extension: | | | | |

Calling Notes: ______________________________________________________________________________________

Email Address: | | | | | | | | | | | | | | | | | | | | | | | | | |

______________________________________________________________________________________

Date of Birth: | | |  **/** | | | **/** | | | | | Age: | | |

MM DD YYYY

Are you a twin? Yes No Gender: Male Female

______________________________________________________________________________________

Are you of Hispanic origin such as Mexican-American, Puerto Rican, or Cuban? Yes No

What do you consider your race to be? White Black Asian

American Indian, Aleutian, Alaska native, or Eskimo

Pacific Islander Don’t Know

Other _____________________________________

Are you a U.S. Citizen? Yes No If no, Visa? __________________ No Answer

What is your Marital Status? Never Married Married Widowed Separated Divorced No Answer

Do you have high blood pressure? Yes No Family history of high blood pressure? Yes No

Do you have diabetes? Yes No Family history of diabetes? Yes No

Have you ever been diagnosed with breast or colon cancer? Yes No Family history of cancer? Yes No

Height: | | ft. | | | in Weight: | | | | lbs BMI: | | |

Has anyone in your family had a heart attack before the age of 50? Yes No

What is your cigarette smoking history? Never Formerly Current

How often do you drink wine, beer, or liquor? Never Daily Once a Week Less than 2 per month

____________________________________________________________________________________________________________

Are you taking any medications (including hormones or contraceptives? Yes No

If yes, what medications are you taking? _________________________

# FEMALES ONLY

Can you still have children? Yes No

If no, why not? Complete Hysterectomy Partial Hysterectomy

Postmenopausal Tubal Ligation

Other; cause ___________________________________

How did you learn of the study? Flyer Radio TV Newspaper Screening Event / Presentation Word of Mouth

Mailout / Brochure Billboard Email / Website Other: _________________________________

Telephone Screening Status: Eligible ( I) Rejected ; Reason for Reject: Heart Disease Diabetes

High Blood Pressure Cholesterol Medications

Diet Eating Disorder

Stroke Cancer

Medical History Other Study

Moving Limited Physical Activity

Recent Pregnancy Other __________________

Interviewer Initials: _________ Interview Date: _______________________

###### Appendix C

Study Specific Questions

# Steps to Increase Physical Activity

# Study Specific Questions

## Adults (Males & Females) BMI **25 – < 35**

**Age 35-64 years**

### Design: 60 Volunteers (30 control group; 30 intervention group) for 3 weeks, 7 days each week.

#### YES NO

1. Do you have heart disease? ____ ____

2. Do you have diabetes? ____ ____

3. Do you have high blood pressure? ____ ____

If so, what medication are you on? **(Hytrin excludes) ____________________________**

4. Are you on cholesterol medication? ____ ____

5. Are you on a medically supervised diet? ____ ____

6. Have you ever been diagnosed with an eating disorder? ____ ____

7. Have you ever had a stroke? ____ ____

1. Have you ever been diagnosed with cancer? ______ ______

9. Have you ever had Hepatitis B, C, or HIV? ____ ____

10. Do you have or ever had tuberculosis? ____ ____

11. Are you currently enrolled in any other study? ____ ____

12. Are you planning to move from area in next 30 days? ____ ____

13. Has your doctor told you to limit physical activity? ____ ____

## Females

14. Have you had a baby recently or are you

planning to become pregnant within the next 30 days? ____ ____

###### Note: With the exception of question 3, to qualify all other answers must be NO.

**OVER →**

**Education (check the highest level completed)**

1. Grades 0-8 
2. Some High School 
3. High School diploma/GED 
4. 1-3 years college, business or technical school 
5. College degree 
6. Post graduate degree 

**What is your present employment status?**

1. Employed full time (at least 36.5 hrs/week) 
2. Employed part-time (at least 20hrs/week) 
3. Retired 
4. Unemployed 

5. Medical disability 

**What is your approximate household income?**

1. Less than $10,000 per year 
2. $10,000 - $19,999 per year 
3. $20,000 - $29,999 per year 
4. $30,000 - $49,999 per year 
5. $50,000 - $69,999 per year 
6. $70,000 - $89,999 per year 
7. $90,000 - $109,999 per year 
8. $110,000 - $139,999 per year 
9. $140,000 per year and above 

**What is the total number of people living in your household?**

1. 1 

2. 2 

3. 3 

4. 4 

5. 5 

6. 6 

7. 7 or more 

###### Appendix D

###### AMPM

###### 24-Hour Recall


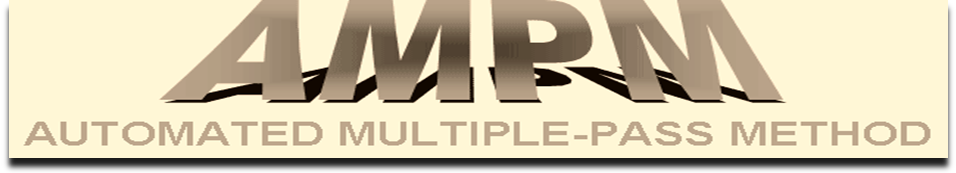


**What is AMPM?**

- Computerized method for collecting interviewer-administered 24-hour dietary recalls either in person or by telephone.
- Research-based, multiple-pass approach employing 5 steps designed to enhance complete and accurate food recall and reduce respondent burden.
- Method used in ***What We Eat in America***, the dietary interview component of the National Health and Nutrition Examination Survey, and other research studies.

**Features of AMPM**

- Employs research-based strategies to enhance dietary recall:
      - Respondent-driven approach allowing initial recall to be self-defined
      - Association with the day's events
      - Probes for frequently forgotten foods
      - Repetition with minimal burden
      - Reviews 24-hour day
      - Placement of foods with eating occasions
- Interviewer-administered, in person or by telephone
- Extensive automated capabilities, including:
      - Unique questions and response options specific for each food
      - Routing of questions based on previous responses
      - Food lookup tables reflecting today's food market
      - Ability to add, change, or delete foods anytime during the interview
      - Automated edit checks performed during data entry
      - Notepad features for interviewer comments
- Companion Food Model Booklet, an aid for estimating portion sizes
- Companion, supportive computer systems for auto-coding, manual coding, and quality control
- Utilizes the Food and Nutrient Database for Dietary Studies

| **5-Step Multiple-Pass Approach** | | |
| --- | --- | --- |
| **Step** |  | **Purpose** |
| **Quick List** |  | Collect a list of foods and beverages consumed the previous day. |
| 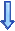 |  |  |
| **Forgotten Foods** |  | Probe for foods forgotten during the Quick List. |
| 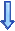 |  |  |
| **Time &  Occasion** |  | Collect time and eating occasion for each food. |
| 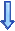 |  |  |
| **Detail Cycle** |  | For each food, collect detailed description, amount, and additions. Review 24-hour day. |
| 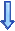 |  |  |
| **Final Probe** |  | Final probe for anything else consumed. |

Read more about USDA's Automated Multiple-Pass Method:

Bliss, R.M. (2004). [Researchers produce innovation in dietary recall.](http://hdl.handle.net/10113/23049)*Agric Res 52*(6):10-12.

Raper N, Perloff B, Ingwersen L, Steinfeldt L, and Anand J. (2004). [An overview of USDA’s Dietary Intake Data System.](http://hdl.handle.net/10113/20984)*J Food Compos Anal 17*(3-4):545-55.

McBride J. (2001). [Was it a slab, a slice, or a sliver? High tech innovations take survey to new level.](http://hdl.handle.net/10113/23006)*Agric Res 49*(3):4-7.

**Information Collected by AMPM**

The AMPM collects a list of all foods and beverages consumed in a 24-hour period. Information captured by AMPM includes:

- For each food... 
      - Description of food
      - Additions to the food (for example, milk on cereal or cream in coffee)
      - Combination code that identifies foods eaten together
        (such as milk added to cereal)
      - Amount of food consumed
      - Time eaten
      - Name of eating occasion
      - Where obtained
      - Eaten at home or not
- Water consumption – bottled and tap water
- Use of salt in preparing foods and at the table
- Whether the amount of food consumed on the recall day was much more than usual, usual, or much less than usual
- Currently on a diet to lose weight or some other health-related reason

**AMPM Applications**

The AMPM is being used in large national surveys in the United States and Canada:

- [***What We Eat in America***, NHANES](http://www.ars.usda.gov/Services/docs.htm?docid=13793) – Yearly since 2002: two recalls each for 5,000 individuals.
- Canadian Community Health Survey, 2004 – 30,000 recalls.
- Other studies in the United States.

**Contact:**

Ms. Alanna J. Moshfegh, Research Leader
USDA/ARS Food Surveys Research Group
BARC-West
10300 Baltimore Avenue Bldg 005 Rm 102
Beltsville, Maryland 20705-2350

[alanna.moshfegh@ars.usda.gov](mailto:alanna.moshfegh@ars.usda.gov)
Phone (301) 504-0170
Fax (301) 504-0376

###### Appendix E

Physical Activity Readiness Questionnaire (PAR-Q)

**
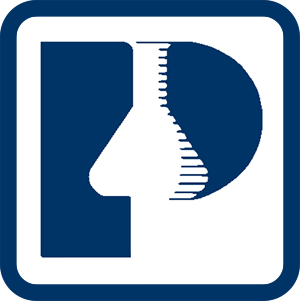
**

**Physical Activity Readiness Questionnaire (PAR-Q)**

For most people, physical activity should not pose any problems or hazard. PAR-Q has been designed to identify the small number of adults for whom physical activity might be inappropriate or those who should have medical advice concerning the type of activity most suitable.

| 1. | Has your doctor ever said you have heart trouble? | Yes | No |
| --- | --- | --- | --- |
| 2. | Do you frequently suffer from pains in your chest? | Yes | No |
| 3. | Do you often feel faint or have spells of severe dizziness? | Yes | No |
| 4. | Has a doctor ever said your blood pressure was too high? | Yes | No |
| 5. | Has a doctor ever told you that you have a bone or joint problem such as arthritis that has been aggravated by exercise, or might be made worse with exercise? | Yes | No |
| 6. | Is there a good physical reason not mentioned here why you should not follow an activity program even if you wanted to? | Yes | No |
| 7. | Are you over age 65 and not accustomed to vigorous exercise? | Yes | No |

**If a person answers yes to any question, vigorous exercise or exercise testing should be postponed. Medical clearance may be necessary.**

Signature Date

Witness Date

Timothy Church, MD Date

###### Appendix F

###### Physical Activity

###### Educational Materials

# Get Moving!

**This educational piece is designed to help you learn:**


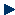
 Benefits to exercising


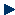
 How to get started with an exercise regime


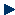
 How to prevent sore muscles or cramps


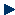
 How to properly stretch


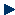
 When to stop exercising


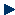
 Understand the F.I.T.T. principle

**Research shows that being more active will:**


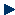
 Help you lose weight and keep it off.


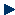
 Help you feel better.


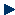
 Make you more physically fit.


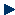
 Make it easier for you to do your daily work, like climbing stairs.


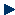
 Lower your risk for some kinds of cancer.


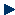
 Lower your risk for heart disease.

Being active:

- Raises HDL cholesterol (the “good” cholesterol in the blood).
- Lowers triglycerides.
- Lowers blood pressure.

**What are the recommendations?**

**For the first time, the USDA Dietary Guidelines for Americans spell out how much physical activity you need. They recommend:**

- **At least 30 minutes of moderate-intensity physical activity, above your usual activity, on most days of the week to reduce the risk of chronic disease in adulthood. Greater health benefits, say the Guidelines, can be reaped with a more intense program or one that is of longer duration.**
- **About 60 minutes of moderate- to vigorous-intensity activity most days of the week to help manage body weight and prevent gradual, unhealthy body-weight gain in adulthood.**
- **At least 60 to 90 minutes of moderate-intensity physical activity daily to sustain weight loss in adulthood.**

Not all ways of being active will strengthen your heart, only those that are “F.I.T.T.”

**F.I.T.T. stands for Frequency, Intensity, Time, and**

**Type of Activity**

|  | **What to do:** |
| --- | --- |
| **Frequency**  (How often you are active) | - Try to be active on **most days of the week**. - Increase slowly. |
| **Intensity**  (How hard you are working while being active: how fast your heart beats) | - Work hard but not too hard. Keep your intensity **similar to a brisk walk.** - Breathe fast enough that you **can talk but not sing.**   If you can break into song, speed it up!  If you have trouble breathing and talking, slow down. |
| **Time**  (How long you are active) | - Stay active for **at least 10 minutes in a row without breaks**. - The total minutes of activity you do per week should be your **activity goal for the week or more.** |
| **Type of activity** | - Do heart fitness activities--those that make your heart work, such as brisk walking, riding your bike, others. - Use large muscle groups (such as legs and arms). - Last 10 minutes or longer. |

**Finding the time**

Many people say, “I would be more active if I could find the time.”

**You *can* find the time to be active.** Here’s how:

- Set aside **one block of time on at least 3 days a week** to be active. Do something you like. For example, get up early and walk the dog before work.
- Look for **short periods of free time (at least 10 minutes)** during the day. Use the time to be active. For example, walk during your coffee break, for part of your lunch hour, and/or between meetings at work.
- Park further away from the store or where you work.

**Make a written plan for every day of the week.** Plan what activity you will do. Plan when and for how long you will do it.

Here’s an example of small activity bouts:

| **Day** | **What I will do** | **When** | **Minutes** |
| --- | --- | --- | --- |
| **Monday** | Walk the dog | 7:00 am | 10 |
| **Tuesday** | Walk during coffee break | 10:00 am | 10 |
| **Wednesday** | Ride a bike | 7:00 am | 10 |
| **Thursday** | Walk during coffee break | 10:00 am | 10 |
| **Friday** | Walk the dog | 7:00 am | 10 |
| **Saturday** |  |  |  |
| **Sunday** |  |  |  |
| **Total minutes for the week:** | | |  |

Aim for the recommended minutes of physical activity per day and record your total for the week. Recording helps you stay on track. Several short activity sessions per day can add up to big benefits!!

**Keep it safe**

Being active is usually quite safe. But in rare cases, problems can arise (such as with running or jogging). The best approach is to prevent problems and keep it safe. Contact your doctor with any questions.

**Prevent sore muscles or cramps.**


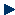
 Only increase how often, how hard, and how long you’re active. a little at a time


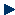
 Wear good and comfortable shoes.


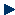
 Drink plenty of water before, during, and after being active.


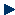
 Wear socks that fit well, are comfortable, and keep your feet dry.

- Warm-up before and cool-down after every activity.

| **Warm-up** (Most warm-ups take only 5 to 15 minutes.)   - Do whatever activity you plan to do but at a lower intensity (lower heart rate) for a brief time. This may mean walking slowly for a few minutes before speeding up. - Do a few minutes of mild stretching if you plan to do something more vigorous than walking.     **Cool-down** (Most cool-downs take only 5 to 15 minutes.)   - Do whatever activity you have been doing, but at a lower intensity (lower heart rate) for a brief time. - Do a few minutes of mild stretching, especially if you’ve done something more vigorous than walking. |
| --- |

**
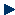
 If you do get a muscle cramp:** Stretch the muscle, then massage it. Repeat.

**
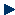
** If there is still pain, put ice on the cramp for a few minutes.

**
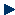
** Then repeat the massage and stretching.

**Stretch those Muscles**

Your body is like a rubber band.

Your muscles will become less flexible as you age and when you are not active. This makes movement more difficult. For this reason, stretching is important.

Stretching is one of the best ways to prevent and avoid muscle soreness, cramps, and hurting yourself. Stretching also helps you be more flexible and feel relaxed.

**How to properly stretch**


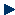
 Do a short warm-up *before* stretching.


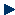
 Move slowly until you feel the muscle stretch. A safe stretch is gentle and relaxing.


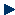
 Hold the stretch steady for 15 to 30 seconds. Do NOT bounce.


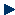
 Relax. Then repeat 3 to 5 times.


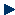
 Stretch within your own limits. Don’t compete.


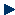
 Breathe slowly in and out. Do NOT hold your breath.

- Relax, enjoy, and feel good about yourself.

**Important**: Never stretch if you have pain before you begin.

If a stretch causes pain, stop doing it.

**How hard are you working?**

Most people have a good sense of how hard they are working when they’re being active. Listen to your body.

Rate yourself on the following scale while you’re being active.

|  |  |  |  |  |  |  |
| --- | --- | --- | --- | --- | --- | --- |
| Very, Very Light? | Very Light? | Fairly  Light? | Somewhat  Hard? | Hard? | Very Hard? | Very, Very Hard? |
| ***Examples:*** | | | | | | |
| “I’m not  working hard  at all. I can talk and even sing easily.” | | “I’m working and breathing a little harder than usual. I can still talk easily.” | “I’m working and breathing somewhat hard. I can talk fairly easily.” | “I’m working hard and breathing deeply. I can still talk.” | “I’m working very hard. I can’t catch my breath or talk.” | |
|  | |  | | |  | |

**Right now:**

- **Stay in the range between “fairly light” and “somewhat hard.”**

Over time, you may be able to progress to the range between “somewhat hard” and “hard.”

**When to stop exercising**

Being active is usually quite safe. But in rare cases, problems can happen.

Be aware of some of the signs and symptoms of when to stop exercising.

- **Chest pain or discomfort**

What: Uncomfortable feeling of pressure, pain, squeezing, or heaviness

Where: Possibly in the:

- Center of the chest,
- Spread throughout the front of the chest, or
- Spreading to the shoulder(s), arm(s), neck, and back

What to do: **Stop and rest.**

- If it doesn’t go away after 2-4 minutes, go to an emergency room or call 911.
- If it does go away, but returns each time you exercise, see your doctor.
- **Unusual shortness of breath, sweating, feeling lightheaded, or feeling sick to your stomach.**

What to do: **Stop and rest.**

- If it doesn’t go away after 2-4 minutes, go to an emergency room or call 911.
- If it does go away, but returns each time you exercise, see your doctor. These may or may not be signs of something serious like a heart problem.

**When exercising, watch for:**

- **Unusual foot pain or numbness**
- **Severe muscle soreness**
- **Joint pain or swelling**
- **Blisters, ulcers, redness or tenderness**

What to do: Call your doctor if these occur.

For now, **start slowly** and **gradually slow down** when you are finished.


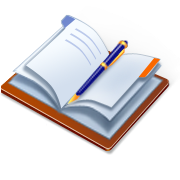
**Next steps**


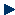
 Record only the time you are *doing* the activity (Don’t include breaks).


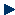
 Achieve your target goals.


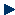
 Walk (or do something like walking) to achieve the physical activity recommendations.

If you have a concern about too much too soon, then start slowly and work up to the recommendation at your own pace**.**

- Include a friend or family member if you like.
- Plan activities you LIKE to do.

Make a **plan for how active you will be** next week:

|  | **What I will do** | **When** | **Minutes** |
| --- | --- | --- | --- |
| Monday |  |  |  |
| Tuesday |  |  |  |
| Wednesday |  |  |  |
| Thursday |  |  |  |
| Friday |  |  |  |
| Saturday |  |  |  |
| Sunday |  |  |  |
| **Total minutes for the week (30 minutes or more):** | | |  |

EXERCISE AND FITNESS WEBSITES

[www.aahperd.org](http://www.aahperd.org/) American Alliance for Health, Physical Education, Recreation, and Dance. Provides links to American Association for Active Lifestyles and Fitness, American Association for Health Education, American Association for Leisure and Recreation, National Association for Girls and Women in Sport, National Association for Sport and Physical Education, and the National Dance Association.

[www.acsm.org](http://www.acsm.org/) American College of Sports Medicine. This is the homepage of the largest, most respected sports medicine and exercise science organization in the world.

[www.acefitness.org](http://www.acefitness.org/) American Council on Exercise. This not-for-profit organization sets certification standards for exercise professionals and runs a consumer fitness hotline offering referrals to ACE certified personal trainers. Their web site features information about their programs, referrals to fitness resources and news about exercise and fitness.

[www.cascade.org](http://www.cascade.org/) This site is loaded with bicycling websites all over the world. Nearly anything you might want to know regarding bicycling can be accessed from this page.

[www.fitnessworld.com](http://www.fitnessworld.com/) This site provides information about exercise books and videos, recent news highlights related to health and fitness, and answers to frequently asked questions about fitness.

[www.ncppa.org](http://www.ncppa.org/) National Coalition for Promoting Physical Activity. This is a premier organization in the country to promote physical activity. The objective of NCPPA is to unite the strengths of public, private and industry efforts into a collaborative partnership to inspire Americans to lead physically active lifestyles to enhance their health and quality of life.

[www.physsportsmed.com](http://www.physsportsmed.com/) The Physician and Sports Medicine Online. This website features abstracts from the current issue of the magazine, actual articles from previous issues, a search feature to find selected topics from back issues, and a Personal Health section with patient-oriented articles on exercise, nutrition and injury prevention.

[www.rrca.org](http://www.rrca.org/) Road Runners Club of America. Novice and experienced runners alike will find a vast array of information provided by this not-for-profit organization, including a calendar of events, guidelines for putting on a race, how to start a club and tips for running safely.

[www.runningpage.com](http://www.runningpage.com/) The Running Page. This site contains information about upcoming races, race results, places to run, running related products, magazines, and other information.

[www.runnersworld.com](http://www.runnersworld.com/) Runner’s World Online. This site contains a wide variety of information about running, including tips for beginning runners, advice about training, and a shoe buyer’s guide.

[www.scoi.com](http://www.scoi.com/) Southern California Orthopedic Institute. This site provides information about a variety of orthopedic problems, including those relating to back and joint injuries.

[http://fiat.gslis.utexas.edu](http://fiat.gslis.utexas.edu/) An excellent jump site to women’s sports pages around the www. Some of the topics include women’s sports, organizations, and issues in women’s sports.

[www.dstc.edu](http://www.dstc.edu/) This website is devoted to a collection of abdominal fitness FAQs on shaping your abdomen.

[http://sln.fi.edu](http://sln.fi.edu/) This site is all about how getting started with an exercise program. It also includes info on selecting a fitness center, exercise equipment, guidelines for beginners, and different kinds of exercises.

[www.fitnessonline.com](http://www.fitnessonline.com/) This site provides info on exercising and proper nutrition. It also has an online trainer as well as fitness calculators.

[www.womenfitness.net](http://www.womenfitness.net/) An online guide to healthy living and optimal fitness for women. This site also provides useful fitness tools to use in addition to articles to read.

[http://exercise.about.com](http://exercise.about.com/) This site provides extensive info on exercise and lots of resource info including a personal trainer on the Internet.


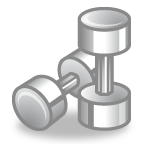


Study Forms

***USING YOUR PEDOMETER***

# Getting Started

|  | 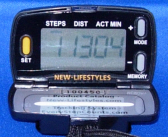 |  |
| --- | --- | --- |

**Beginning tomorrow:**

Tomorrow, put your pedometer on, reset it to zero, and go about your day without changing a thing. This is very important so that you can establish your usual, or baseline, activity. Write down the number of steps you take that first day here: ___________

**Figuring out your steps:**

Now, let’s figure out how many steps you take in 10 minutes of continuous walking. So, put your pedometer on, reset it to zero, check the time on your watch, and walk at your normal pace for 10 minutes. How many steps do you take in 10 minutes? ___________________

A good rule of thumb is that 1000 steps/minute taken in 10 minutes is equivalent to walking at a moderate intensity pace. Look at your number and determine whether you need to walk a bit faster to meet the moderate intensity recommendation.

**How many steps are enough?**

A good guideline is to add 60 minutes of moderate intensity activity, like brisk walk, to your usual daily activities. This is equivalent to an additional 6000 steps. But it also depends on your baseline activity.

- If you take less than 5,000 steps/day at baseline, add 6,000 steps/day
- If you take between 5,000 and 7,500 steps/day at baseline, add 3,000 steps/day
- If you already take more than 7,500 steps/day at baseline, consider adding more steps/day and/or walking faster.

**Setting your goal**

Add the amount of steps you plan to increase by_____________ to your baseline steps________________. This is your step goal equivalent to public health guidelines for physical activity. Now wear your pedometer and try to hit this target every day. The most important strategy is to look at your pedometer frequently throughout the day and see how you are doing. Also be sure to write down the steps you take each day on the calendar we gave you.

**Strategies for increasing your physical activity easily:**

- park farther from the office entrance
- take the stairs (up and/or down) instead of elevators
- walk to lunch
- take the dog for a walk
- go for a walk in the evenings with your spouse
- leave the car at home and walk to the convenience store
- walk around the shopping mall

***Attached are some instructions for recording your minutes of physical activity. Please review these instructions and complete the calendar by recording as indicated.***

Written Instructions

Pedometer

# INSTRUCTIONS

**Pedometer:** The pedometer counts your steps taken. This pedometer resets automatically at midnight each night. You do not need to reset it. Don’t worry if it the screen reads zero when you put it on in the morning.

Wear your pedometer during your waking hours. Do not wear it in the shower or bath, or while swimming.

**Every morning**, when you put the pedometer on:

Write down the date of the day, and the time you put your pedometer on in the calendar we gave you.

Then clip the pedometer to your waistband on the front of your body near your hipbone. You can wear the pedometer under your clothes or on the outside of your waistband if you wish. Remember to use your safety clip!

**Every night when you get ready for bed**, take the pedometer off and open it. On your calendar, write down the time you took the pedometer off. Write down the number of steps shown on the screen on your daily activity calendar. YOU DO NOT NEED TO RESET IT.

Circle Y=”YES”, or N=”NO” in answer to each of the questions on your calendar.

For example, Were you injured or sick today?

Did you go to work today?

Did you do any sports today?

Did you exercise today?

If you did sports or exercise, write down the type of activity you did, and how much time you were active.

For example,

Walk 30 minutes

## ***Tennis 15 minutes***

# If you forget to wear the pedometer one day, just leave the day blank

Thank you for your time!

If you have any questions, please call *********

Calendar

Pedometers

Written Instructions

Accelerometers

# INSTRUCTIONS

**Accelerometer:** The accelerometer keeps track of time you are active and inactive.

Wear your accelerometer on the elastic belt at your waist near your hipbone (in line with your arm pit) during your waking hours. Do not wear it in the shower or bath, or while swimming.

**Every morning**, when you put the accelerometer and belt on:

Write down the date of the day, and the time you put your accelerometer on in the calendar we gave you.

**Every night when you get ready for bed**, take the belt off. On your calendar, write down the time you took the accelerometer off. YOU DO NOT NEED TO RESET IT OR TAMPER WITH IT IN ANYWAY.

Circle Y=”YES”, or N=”NO” in answer to each of the questions on your calendar.

For example, Were you injured or sick today?

Did you go to work today?

Did you do any sports today?

Did you exercise today?

If you did sports or exercise, write down the type of activity you did, and how much time you were active.

For example,

Walk 30 minutes

## ***Tennis 15 minutes***

# If you forget to wear the accelerometer one day, just leave the day blank

Thank you for your time!

If you have any questions, please call *********

Calendar

Accelerometer

**Steps to Increase Physical Activity**

**Post-Study Survey**

**Study Number:**

**Last Name:** | | | | | | | | | | | | | | | | | | | |

**First Name:** | | | | | | | | | | | | | | | | | **Middle Initial** | |

1. **The following list includes some things that might interfere with or prevent you from exercising or being physically active. For each one, record how often it interferes or prevents you from exercising or being physically active.**

**Some Very**

**Never Rarely times Often Often**

a. Others discourage me. 1 2 3 4 5

b. I am self-conscious about my looks. 1 2 3 4 5

c. I am afraid of injury. 1 2 3 4 5

d. I don’t have time. 1 2 3 4 5

e. I am too tired. 1 2 3 4 5

f. I don’t have a safe place to exercise. 1 2 3 4 5

g. I have no child-care assistance. 1 2 3 4 5

h. The weather is bad. 1 2 3 4 5

i. I am not in good health. 1 2 3 4 5

j. I don’t have the energy to exercise. 1 2 3 4 5

k. I get plenty of exercise at my job. 1 2 3 4 5

l. I don’t have the motivation to exercise. 1 2 3 4 5

m. I don’t like to exercise. 1 2 3 4 5

n. I have no one to exercise with. 1 2 3 4 5

o. I’m just not interested. 1 2 3 4 5

p. I don’t enjoy exercise, sports, or physical activity. 1 2 3 4 5

**Some Very**

**Never Rarely times Often Often**

q. I don’t have equipment. 1 2 3 4 5

r. I don’t have access to facilities or space to exercise. 1 2 3 4 5

s. I don’t have the right skills. 1 2 3 4 5

t. I don’t have enough information or knowledge. 1 2 3 4 5

u. Cost is a barrier. 1 2 3 4 5

v. I don’t have enough self-discipline. 1 2 3 4 5

**2. Please indicate which of the following responses best applies to you, your neighborhood, and the amount of support for exercising that you get from others.**

**Strongly Somewhat Somewhat Strongly**

**disagree disagree agree agree**

**Infrastructure for walking and cycling**

1. There are sidewalks on most of the streets in

my neighborhood. 1 2 3 4

1. The sidewalks in my neighborhood are well

maintained (consider cracks and evenness). 1 2 3 4

1. There are bicycle or walking trails in or near my

neighborhood that are easily accessible. 1 2 3 4

1. The streets in my neighborhood are

hilly making my neighborhood difficult to walk in. 1 2 3 4

**Neighborhood surroundings**

1. There are many attractive natural sites in my

neighborhood (such as landscaping, views). 1 2 3 4

**Neighborhood safety**

1. There is so much traffic along the street I live on

that it makes it difficult or unpleasant to walk in

my neighborhood. 1 2 3 4

1. Most drivers exceed the posted speed limits while

driving in my neighborhood. 1 2 3 4

1. My neighborhood streets are well lit at night. 1 2 3 4
2. The crime rate in my neighborhood makes it unsafe

to go on walks during the day. 1 2 3 4

1. The crime rate in my neighborhood makes it unsafe

to go on walks at night. 1 2 3 4

**Strongly Somewhat Somewhat Strongly**

**disagree disagree agree agree**

1. When walking in my neighborhood there are a

lot of exhaust fumes (such as from cars, buses or

factories). 1 2 3 4

1. There are many unattended dogs in my neighborhood. 1 2 3 4
2. I see a lot of people walking and biking in my

neighborhood. 1 2 3 4

**Social Support**

1. If I had someone like a friend or family member

to exercise with, chances are that I would exercise

more. 1 2 3 4

1. My friends encourage me to exercise. 1 2 3 4
2. I have at least one friend who would commit to

exercise with me. 1 2 3 4

1. Relatives encourage me to exercise. 1 2 3 4
2. I have at least one relative who would commit to

exercise with me. 1 2 3 4

1. When walking in my neighborhood there are a

lot of exhaust fumes (such as from cars, buses or

factories). 1 2 3 4

1. There are many unattended dogs in my neighborhood. 1 2 3 4
2. I see a lot of people walking and biking in my

neighborhood. 1 2 3 4

3. What is the one thing that makes it hardest for you to be physically active?

4. What did you like most about the study's program?

5. What did you like least about the study's program? What do you think should be changed?

6. Would you recommend this program to one of your friends? Why or why not?

**FOR PEDOMETER GROUP ONLY**

7. What did you like most about the pedometer?

8. What did you like least about the pedometer?
